# A Reduction in Ribonucleotide Reductase Activity Slows Down the Chromosome Replication Fork but Does Not Change Its Localization

**DOI:** 10.1371/journal.pone.0007617

**Published:** 2009-10-28

**Authors:** Ingvild Odsbu, Morigen, Kirsten Skarstad

**Affiliations:** Department of Cell Biology, Institute for Cancer Research, The Norwegian Radium Hospital, Oslo University Hospital, Oslo, Norway; University of Massachusetts, United States of America

## Abstract

**Background:**

It has been proposed that the enzymes of nucleotide biosynthesis may be compartmentalized or concentrated in a structure affecting the organization of newly replicated DNA. Here we have investigated the effect of changes in ribonucleotide reductase (RNR) activity on chromosome replication and organization of replication forks in *Escherichia coli*.

**Methodology/Principal Findings:**

Reduced concentrations of deoxyribonucleotides (dNTPs) obtained by reducing the activity of wild type RNR by treatment with hydroxyurea or by mutation, resulted in a lengthening of the replication period. The replication fork speed was found to be gradually reduced proportionately to moderate reductions in nucleotide availability. Cells with highly extended C periods showed a “delay” in cell division i.e. had a higher cell mass. Visualization of SeqA structures by immunofluorescence indicated no change in organization of the new DNA upon moderate limitation of RNR activity. Severe nucleotide limitation led to replication fork stalling and reversal. Well defined SeqA structures were not found in situations of extensive replication fork repair. In cells with stalled forks obtained by UV irradiation, considerable DNA compaction was observed, possibly indicating a reorganization of the DNA into a “repair structure” during the initial phase of the SOS response.

**Conclusion/Significance:**

The results indicate that the replication fork is slowed down in a controlled manner during moderate nucleotide depletion and that a change in the activity of RNR does not lead to a change in the organization of newly replicated DNA. Control of cell division but not control of initiation was affected by the changes in replication elongation.

## Introduction

DNA synthesis in rapidly growing bacteria requires that the replication enzymes recruit and utilize deoxyribonucleotides (dNTPs) at a rate of 10 000–20 000 nucleotides per second (about 1600 nucleotides at each of 6–14 forks, see Materials and Methods). It has been proposed that the local concentration of dNTPs at the replication forks is higher than elsewhere in the cells and that this is achieved by a co-organization of nucleotide synthesizing enzymes and replication enzymes into structures in which intermediates are channeled from one enzyme to the next without diffusion of these intermediates into the surrounding cytoplasm [1], [2]. It has also been suggested that the presence of the structures is dependent on function [3]. Evidence for structures containing both replication enzymes and enzymes involved in nucleotide biosynthesis have been found in phage T4 infected *E. coli* cells during synthesis of viral DNA [4], [5]. Evidence for such structures has so far not been found in uninfected *E. coli* cells.

Ribonucleotide reductase (RNR) is an essential enzyme responsible for the reduction of ribonucleoside diphosphates (NDPs) to deoxyribonucleoside diphosphates (dNDPs) and is the rate-limiting step in nucleotide biosynthesis [6]. The main RNR protein expressed during aerobic growth is a class Ia RNR consisting of two homodimeric subunits encoded by the *nrdA* (R1) and *nrdB* (R2) genes [6], [7]. Both protein activity and gene transcription is under tight regulatory control in order to provide a balanced pool of dNTPs for DNA replication and repair. The R1 subunit contains both the allosteric sites for regulation and the catalytic site for NDP reduction, while the R2 subunit provides the metallo-cofactor (a tyrosyl radical) necessary for reduction of the substrate. Transcription of the *nrdAB* genes is regulated via several types of binding sites near the promoter [7], [8]. These include binding sites for the initiator protein, DnaA, and for the repressor of several ribonucleotide reductase genes, NrdR [7], [9]. It has been suggested that prior to initiation of replication the ATP-bound form of DnaA acts as a repressor of *nrdAB* gene expression. During replication ATP-DnaA is converted to ADP-DnaA which reduces the extent of repression. The conversion of ATP-DnaA to ADP-DnaA thus seems to couple the expression of *nrdAB* to chromosome replication [9]. It has also been suggested that since DnaA can bind dATP [10], and the autoregulation of *nrdAB* is dependent on DnaA, the regulation of *nrdAB* expression by DnaA may also be directed by cellular dNTP concentrations [9]. Also the NrdR protein binds ATP and dATP [11]. It is therefore possible that both proteins function as sensors of dATP levels and regulate *nrdAB* expression accordingly.

Cells containing a temperature-sensitive NrdA protein were found to be able to complete one round of replication at the non-permissive temperature when protein synthesis was inhibited and it was proposed that this was due to protection of the mutant protein by a replication hyperstructure [12]. The same mutant was also found to generate stalled replication forks at the permissive temperature that was not due to the limited supply of dNTPs, but rather assumed to be due to the presence of a less efficient replication hyperstructure causing frequent replication pauses [13].

The SeqA protein binds to hemimethylated GATC sequences present on newly replicated DNA and multimerizes into a left-handed helical filament that probably has a role in organizing the newly replicated DNA [14]–[18]. Such SeqA structures can be visualized *in vivo* as a GFP-fusion and in fixed cells by immunostaining with SeqA antiserum [19], [20]. The SeqA structure could also have a role in stabilizing the proposed replication hyperstructure by functioning as an assembly site [21]. A changed pattern of SeqA structures could therefore be expected if one of the components in the hyperstructure is missing or not functioning properly.

Rapidly growing *E. coli* cells replicate the single chromosome with overlapping cycles [22]. The larger the difference between the generation time and the duration of C (replication) and D (segregation) periods, the larger the overlap [23]. Studies of replication fork localization by comparing the distribution of SeqA structures with the distribution of replication forks in rapidly growing cells indicate that on average 2.5 replication forks are co-localized in a SeqA structure (for instance, in cells with 12 replication forks, the forks are localized to 4 sites in some cells and 6 sites in others) [15], [24], [25]. It was found that cells with impaired function of thymidylate synthase (ThyA) seemed to exhibit a changed pattern of replication fork organization [24]. One explanation for this could be that the structurally altered ThyA protein caused a less stable hyperstructure. Based on this finding and the proposed presence of RNR in the replication hyperstructure described above, we decided to investigate fork organization in cells experiencing a change in the activity or function of the RNR protein.

We investigated three different situations of limiting dNTPs: i) wild type cells incubated with hydroxyurea (HU), ii) cells with different concentrations of ectopically expressed mutant RNR protein, *nrdB* (D367G), and iii) cells with a severely impaired temperature-sensitive RNR protein, *nrdA* (L89P). It was found that the replication process is flexible and can tolerate a considerable slowing of replication fork movement before replication forks stall. The organization of replication forks was not affected by large changes in the activity of RNR protein.

## Materials and Methods

### Bacterial strains, plasmids and growth conditions

All strains used were *E. coli* K-12. Strains and plasmids are listed in Table 1. Cells were grown in AB minimal medium [26] supplemented with 1 µg/ml thiamine, 0.2% glucose and 0.5% casamino acids (glu-CAA medium). Derivatives of MG1655 and *thyA* mutants (E101 and IO08) were supplemented with 100 µg/ml uridine (glu-CAA-uri) and 10 µg/ml thymidine (glu-CAA-thy), respectively. The appropriate antibiotics were added in the following concentrations: 50 µg/ml ampicillin, 50 µg/ml kanamycin and 10 µg/ml tetracycline. Mass growth was monitored by measuring the optical density at 450 nm with a spectrophotometer.

**Table 1:** Bacterial strains and plasmids.

Strains	Relevant genotype	Source/reference
MG1655^a^	F^−^ λ^−^ *rph-1*	[47], [48]
LJ52^b^	MG1655 *attB*::p*nrdA-lacZ*	This work, [27]
SMG367	MG1655/pDSW204	Stèphanie Gon
SMG386	MG1655Δ*nrdAB*/pIO5	Stèphanie Gon
E101^c^	*nrdA1* (ts) *thyA6 deoB37 deoC1 thr-1 leuB6 fhuA21 lacY1 glnV44 λ^−^ rfbC1 rpsL67 thi-1*	CGSC# 5146
IO08	E101/pIO5	This work
HC123^c^	*dnaN159* (ts)	CGSC# 6844
MOR237	MG1655 *recA56 srl*300::Tn10	This work
**Plasmids**		
pDSW204	Promoter down mutation in −35 of pTrc99A	[49]
pIO5^d^	pDSW204-*nrdAB* (pSMG7) with mutation in *nrdB* (D367G))	[50]

a Provided by Jon Beckwith.

b In the experiment with HU (Figure 2, Table 2) the LJ52 strain was used. It is referred to as MG1655 for simplicity.

c Obtained from The Coli Genetic Stock Center, Yale University.

d The *nrdAB* gene on the plasmid pSMG7 [50] used in this study was found to contain a point mutation in *nrdB* that leads to an amino acid change at position 367 (D367G). The plasmid is referred to as pIO5 in the text.

### Calculation of nucleotide incorporation

Duplication of a 4.6 Mb pair genome requires incorporation of 9.2 Mb to synthesize two daughter strands. Since one round of replication takes approximately 50 minutes (Table 2) the replication rate will be 9 200 000 nucleotides/3000 sec = 3067 nucleotides/sec, i.e. each fork during bidirectional replication processes 1533 nucleotides/sec and each polymerase 767 nucleotides/sec (approximately 800 nucleotides/sec). During fast growth cells contain from 6–14 replication forks, i.e. 10 000–20 000 nucleotides are processed per second.

**Table 2:** Cell cycle parameters of MG1655 cells grown in the absence or presence of 5 mM.

Strain	τ^a^	DNA content^b^	Cell mass^b^	DNA/mass^b^	*oriC/terC* c	C+D^d^	C^e^	D^f^	Average no of forks/cell^g^	Average no of SeqA foci/cell	Forks/focus
MG1655	27	1	1	1	3.7±0.4	76	49±5	27	10.2	4.1	2.5
MG1655 5 mM HU	27	1.1	1.3	0.9	5.7±0.3	81	62±4	19	12.7	3.9	3.3

HU.

a τ is the generation time in minutes from one representative cell culture used in the flow cytometry and immunostaining experiments.

b Average DNA content, cell mass and DNA per mass were derived from flow cytometry measurements from the cell culture in **a.** as described in Materials and Methods. All values are relative to MG1655 grown without HU.

c The *oriC/terC* ratio was determined by Southern blot analysis and quantitative PCR as described in Materials and Methods and is the average from 2–5 independent experiments. The standard deviation is included.

d The C+D period (in minutes) was determined from the DNA histogram of cells treated with rifampicin and cephalexin and the generation time as described in Materials and Methods.

e The C period (in minutes) was found from the formula *oriC/terC* = 2^C/τ^. The average τ- value from 2–5 independent experiments was used in the calculation.

f The D period (in minutes) was found by subtraction of C from C+D.

g For calculation of average number of forks per cell, see Materials and Methods and legend to Figure S1.

### P1 transduction

The *attB*::p*nrdA-lacZ* fusion was transferred by P1 transduction from the strain BOS7 [27] to strain MG1655. Positive transductants were selected for by kanamycin resistance.

### Sequencing of the mutant *nrdA* allele

The *nrdAB* operon of the mutant strain, E101, was amplified by PCR using the following primers: 5′- GTGTTCTAAGCAGCTTCCCG–3′ and 5′- ACCTTCGCGACACTGGTACTC–3′. Sequencing of the resultant PCR product was performed at GATC Biotech, Germany.

### Flow cytometry analysis

Exponentially growing cells were harvested at OD_450_ = 0.15 or treated with 300 µg/ml rifampicin and 10 µg/ml cephalexin for 4–5 generations. Rifampicin inhibits new initiations while ongoing forks are allowed to finish and cephalexin inhibits cell division. Thus after drug treatment, cells will contain fully replicated chromosomes of number 2^n^ (n = 0, 1, 2…) if they display synchronous initiation [28] and the number of chromosomes will correspond to the number of origins present in the cell population at the time of drug action. After harvesting, the cells were washed once in ice-cold, filtered TE-buffer and resuspended in 100 µl of the same buffer. Then 1 ml of ice-cold, filtered 77% ethanol was added for fixation. The fixed cell samples could be kept at 4°C for several months. For flow cytometry analysis, 1 ml of fixed cells was washed in 1 ml ice-cold, filtered 0.1 M PB-buffer (pH 9) and stained overnight at 4°C with 1.5 µg/ml fluorescein isothiocyanate (FITC) in the same buffer. The following day, cells were washed in 1 ml ice-cold, filtered 0.02 M TBS-buffer (pH 7.5) and stained with 1.5 µg/ml Hoechst 33258 in the same buffer. Staining of the DNA standard was performed as described previously [29]. Flow cytometry analysis was carried out using a LSRII instrument (Becton Dickinson) equipped with an Argon ion laser and a Krypton laser (both Spectra Physics). The flow data was analyzed using WinMDI software (http://facs.scripps.edu/software.html). The average DNA content and average cell mass per cell were determined as average Hoechst and FITC fluorescence per cell, respectively. The average DNA per mass was found by dividing average DNA content by average cell mass.

### Cell cycle analysis

The cell cycle was analyzed as described previously [23], [25]. From the run-out histogram of cells treated with rifampicin and cephalexin, the fraction of cells that had not initiated replication at the time of drug action (F) was found. The initiation age in minutes (α_i_) was found using F and the theoretical age distribution in the formula: F = 2−2^((τ−αi)/τ)^, where τ is the generation time in minutes. From the initiation age, the generation time and the number of generations spanned per cell cycle, the C+D period was calculated. The C period (replication period) was found from the formula *oriC*/*terC* = 2^C/τ^ [30] where the *oriC*/*terC* ratio was determined by Southern blot analysis and quantitative PCR as described below. The D period (the time between the end of a round of replication and cell division) was found from the C+D and C period ((C+D)−C). The termination age (α_t_) was obtained from the length of the C period. From these data a schematic diagram of the cell cycle could be drawn. A verification of the cell cycle parameters was performed by calculating a theoretical DNA distribution from the values (see supplementary information, Figure S1) and checking that the theoretical distribution fit with the experimentally obtained DNA histogram of exponentially growing cells. The average number of replication forks per cell was calculated from the fractions of cells containing the different numbers of replication forks obtained from the replication fork distribution. For further explanation see Figure S1.

### Southern hybridization

To isolate total cellular DNA, 50 ml of exponentially growing cells (OD_450_ = 0.15) were lysed by incubation at 60°C for 10 minutes in the presence of 1.4% SDS and 4 mM EDTA. The heated mixture was cooled on ice and the DNA was precipitated by addition of 0.2 M NaCl and 0.7 volume of isopropanol. Precipitated DNA was collected by centrifugation and resuspended in TE-buffer, then treated with RNase (100 µg/ml) and extracted twice with phenol, then twice with chloroform. Finally, the DNA was precipitated again in NaCl and ethanol and collected by centrifugation. The DNA pellet was washed in 80% and 96% EtOH and resuspended in TE-buffer. To determine the *oriC/terC* ratio, 1 µg of purified DNA was cut with SalI and XhoI (New England Biolabs Inc.) which gives *oriC*- and *terC*-containing fragments of 6.5 and 1.6 kb, respectively, run on an 1% agarose gel, transferred to a Hybond-N^+^ membrane (Amersham Biosciences) and probed with *oriC* and *terC*-specific ^32^P-labelled probes as described previously [31]. The membrane was scanned on a Storm 840 phosphorimager (Molecular Dynamics) and quantification of radioactive bands was done using ImageQuant software (Molecular Dynamics). Hybridization signals were normalized to the signals of MG1655 wild type cells treated with rifampicin and cephalexin where *oriC* and *terC* bands are present in a 1∶1 ratio.

### Quantitative PCR

Purified chromosomal DNA was digested with *Eco*RI (New England Biolabs Inc.) and 10 ng DNA was used for PCR amplification (7500 RealTime PCR system, Applied Biosystems). The primers used were 5′- GAGAATATGGCGTACCAGCA-3′ and 5′- AAGACGCAGGTATTTCGCTT -3′ for *gidB* and 5′- TCCTCGCTGTTTGTCATCTT -3′ and 5′- GGTCTTGCTCGAATCCCTT–3′ for *ter*. The fluorescent probes were 5′ FAM- CAACCTGACTTCGGTCCGCG–TAMRA 3′ for *gidB* and 5′ FAM - CATCAGCACCCACGCAGCAA–TAMRA 3′ for *ter*. The data from the samples were normalized to the data obtained from MG1655 wild type cells treated with rifampicin and cephalexin where *oriC* and *ter* regions are present in a 1∶1 ratio.

### Immunostaining with SeqA antibody and fluorescence microscopy

Cells were prepared for immunofluorescence microscopy and visualized as previously described [32].

### Western blotting

Exponentially growing cells were harvested at OD_450_ = 0.15 and cell extracts were prepared as described previously [29]. The same amount of total protein from each strain or treatment was subjected to 8 or 10% SDS-PAGE and proteins were transferred to a polyvinylidene difluoride (PVDF) membrane by semi-dry blotting. The membrane was probed with NrdA, NrdB or SeqA polyclonal antibodies. Detection was performed with ECF Western Blotting kit (GE Healthcare) and the membrane was scanned on a ChemiGenius instrument (Syngene). Quantification of fluorescent bands was carried out with ImageQuant software (Molecular Dynamics).

### dNTP pool size measurement

Nucleotides were extracted from the cells as previously described with some modifications [33]. Cells were grown in glu-CAA-uri medium to OD_450_ = 0.15 followed by filtration of 300 ml cell culture through 47 mm, 0.45 µm nitrocellulose filter (Millipore). After filtration the filter was placed cell-side down in a beaker containing 4 ml ice-cold 1 M HCOOH and kept for 30 minutes on ice. Then the filter and extract were transferred to a 50 ml tube and vortexed to remove all cells from the filter. The extract was centrifuged and the supernatant was completely dried in a Savant AES1010 SpeedVac to remove the formic acid. The pellet was resuspended in 200 µl 0.05 M NH_4_H_2_PO_4_ pH 3.4, filtered and injected into a Partisphere SAX column (4.6 mm×250 mm, Whatman). HPLC was performed with an Agilent 1100 system and detection was at 254 nm. The gradient used was a modification of the one used by Buckstein and co-workers [34]. A linear gradient of 100∶0 (A∶B) to 56∶44 was run over 20 minutes followed by a linear gradient of 56∶44 to 50∶50 over the next 10 minutes and finally a linear gradient to 0∶100 from 30–45 minutes. The flow rate was 1 ml/min. Buffer A and B consisted of 0.05 M and 1 M NH_4_H_2_PO_4_ pH 3.4, respectively.

The column and gradient used separated all the dNTPs from the corresponding NTPs. However, due to the low concentrations of dNTPs in the cell extract, the peak containing dTTP co-eluted with ATP. In order to measure the amount of dTTP, the dNTPs were separated from the NTPs via a boronate affinity column as previously described [11]. The fraction containing only dNTPs was analyzed further with HPLC. As a positive control the pool size of wild type cells overproducing RNR was measured and showed increasing amounts of all the dNTPs relative to wild type cells (data not shown).

### UV treatment

MG1655 wild type cells were grown exponentially in glu-CAA-uri medium at 37°C to OD_450_ = 0.15 and then UV irradiated with 50 J/m^2^ in a Petri dish while stirring. Samples were taken at 0, 15, 30, 60 and 90 minutes after irradiation and fixed in 70% ethanol.

## Results

### Moderate inhibition of RNR activity led to extension of the C period and a higher number of replication forks

**Figure 1:**
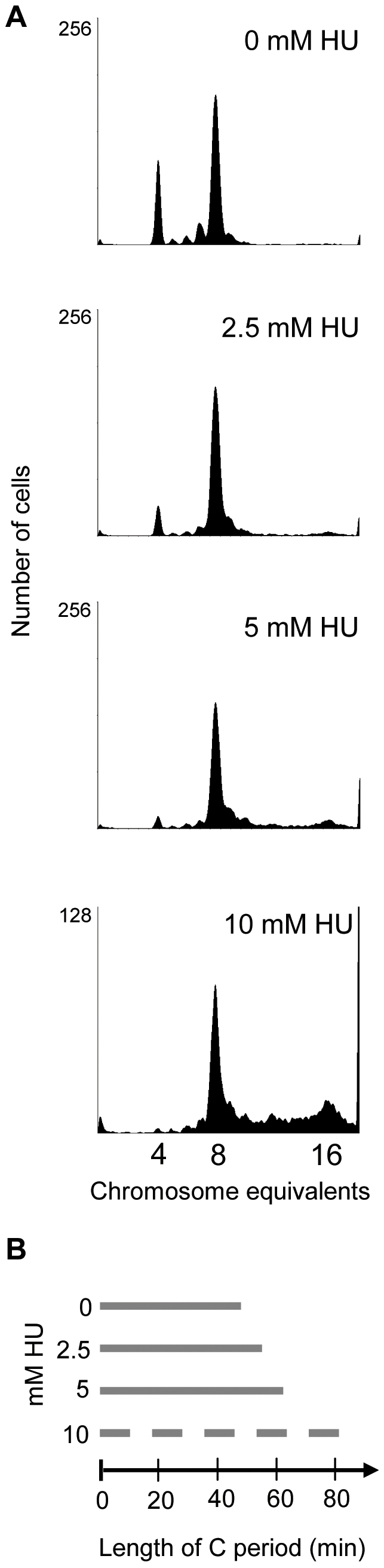
The duration of C increased with increasing HU concentration. A: Cells were grown exponentially in glu-CAA-uri at 37°C in the presence of 0, 2.5, 5 or 10 mM HU for about eight generations to OD_450_ = 0.15, then fixed in 70% ethanol after incubation for 4–5 generation times in rifampicin and cephalexin. The number of cells is plotted against the DNA content. B: The durations of the C periods obtained by growth with the different HU concentrations. C periods were found from the *oriC/terC* ratio obtained by quantitative PCR and Southern blot analysis of cells used in A (see Materials and Methods). The C period of cells grown with 10 mM HU is drawn as a broken line because in this case the C period is aberrant due to replication fork stalling and reversal.

Moderate concentrations of hydroxyurea (HU) were used to partially inhibit the activity of ribonucleotide reductase (RNR). HU inhibits the activity of RNR by scavenging its free radical necessary for catalysis, and the reduced RNR activity leads to decreased concentration of dNTPs in the cell [35]. Here, wild type cells (MG1655) grown in glu-CAA-uri medium at 37°C, were treated with different concentrations of HU (2.5, 5 and 10 mM) to see if we could obtain a situation of reduced fork movement, but unchanged growth rate. Flow cytometry analysis revealed that cells initiated earlier in the cell cycle with increasing concentrations of HU (Figure 1A). This can be seen from the run-out histograms by the increase in the numbers of origins per cell with increasing HU concentration. When an exponentially growing cell culture is treated with rifampicin and cephalexin, new initiations and cell division are inhibited while ongoing rounds of replication are allowed to finish. Thus the number of completed chromosomes corresponds to the number of origins at the time of drug action. If initiation takes place earlier in the cell cycle, this will be reflected as an increase in the fraction of cells with the highest number of completed chromosomes in the run-out histogram. Whereas wild type cells contained four and eight origins, cells treated with 2.5 mM or 5 mM HU contained mainly eight, and cells treated with 10 mM HU, eight and sixteen origins (Figure 1A). The replication period (C), found from the *oriC/terC* ratio obtained by Southern blot analysis and quantitative PCR, increased by about 15%, 30% and 70% when grown in 2.5 mM, 5 mM and 10 mM HU, respectively (Figure 1B, Table S1). The increased C period obtained after HU-treatment could be explained by a reduced rate of fork movement in general or by frequent replication fork stalling causing an extended replication period due to repair of stalled forks. If forks stall more frequently in the presence of HU, cells deficient in one of the repair enzymes necessary for fork reconstitution should be more sensitive to HU-treatment. The survival of MG1655 *recA* ^−^ cells grown in the presence of 2.5 mM or 5 mM HU was found to be 100%. This shows that cells can tolerate 5 mM HU without an increase in the frequency of fork stalling. In the presence of 10 mM HU the survival of cells in a *recA* ^−^ background has been reported to be about 10^−3^ relative to wild type cells grown under the same conditions [36]. This indicates that in cells grown in the presence of 10 mM HU replication forks frequently stall. This is apparent also in the run-out histogram of cells grown in this concentration of HU (Figure 1A, bottom panel), where DNA contents are found also between the integer values of 8 and 16 chromosome equivalents, indicating the presence of incomplete chromosomes. In the study mentioned above it was also reported that 10 mM HU induced a need for the RecBC, RuvABC and RecG proteins in addition to RecA [36]. These proteins act on double-stranded ends formed by replication fork reversal [37]. It is thus possible that HU-treatment provokes replication fork reversal. It has also been reported that in the presence of high HU concentrations (>50 mM) it is likely that the Y-family DNA polymerases play a major role in DNA replication [38]. We wished to study a situation with an increased C period, but no (or a normal amount of) fork stalling, and therefore chose the 5 mM HU condition for detailed study.

**Figure 2:**
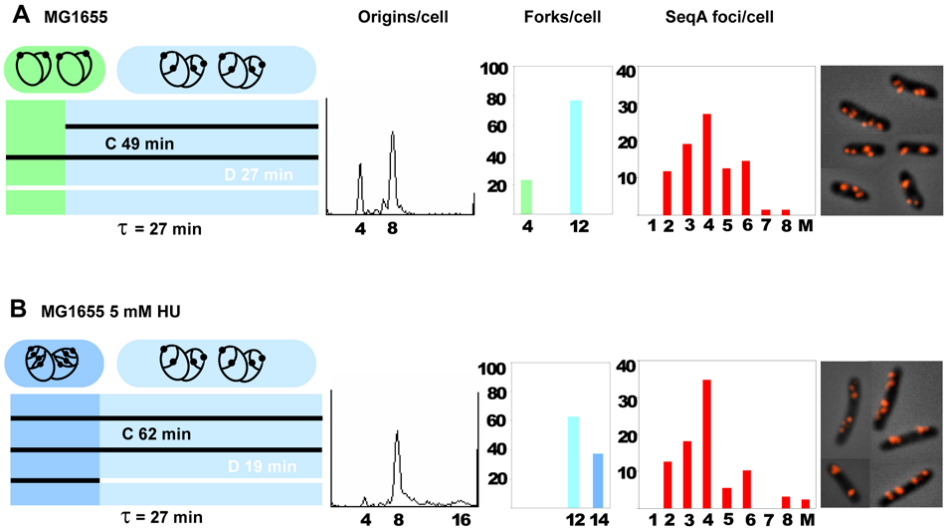
Replication patterns, fork- and SeqA focus distributions in cells grown w/ or w/o HU. MG1655 wild type cells were grown in the absence (A) or presence (B) of 5 mM HU (cells from panel 1 and 3 in Figure 1A). First panel: schematic diagram of the cell cycle where the length of the diagram corresponds to the generation time (τ). It also represents the cell age where newborn cells are found to the left and the oldest cells that are about to divide are found to the right. The time periods before and after the different cell cycle events (initiation and termination) are in different colors. On top of the diagram cells with corresponding replication patterns are drawn. A black circle and black dot represent the chromosome and the origin, respectively. Each horizontal bar represents one generation and is colored black for C and white for D as one replication cycle is followed through three generations. The duration of C was found from the *oriC/terC* ratio obtained by Southern blot analysis and quantitative PCR. The initiation time point and C+D period were determined from the run-out histogram and the generation time, and D was determined by subtraction of C from C+D (Figure S1 and Materials and Methods). In A the age of initiation was found to be at about 5 minutes since 23% of the cells contained 4 origins (green). Cells older than 5 minutes contained 8 origins and 12 forks (light blue). Cells grown in the presence of 5 mM HU initiated replication at 4 origins when newborn and thus all cells contained 8 origins (Figure 2B, left panel). When young, these cells contain 14 replication forks (blue), and after termination of the two “oldest” forks the cells contained 12 forks (light blue). Second panel: DNA histograms of rifampicin- and cephalexin treated cells. Third panel: the distributions of replication forks in exponentially growing cells where the percentages of cells are plotted against the numbers of replication forks per cell. The different colors correspond to the different stages of the cell cycle as used in the diagram in the first panel. Fourth panel: SeqA focus distributions in exponentially growing cells where the percentages of cells are plotted against the numbers of SeqA foci per cell. A: 136 cells counted, the foci number of 11.8% of the cells was not determined. B: 127 cells counted, the foci number of 13.4% of the cells was not determined. Fifth panel: micrographs of fixed cells immunostained with antibody against SeqA.

A comparison of the replication patterns of cells grown in the absence or presence of 5 mM HU showed that the HU-treated cells initiated replication when newborn whereas the untreated cells initiated at age 5 minutes (Figure 2), and also showed that the C period extension seen in the HU-treated cells was compensated by a reduction in the D period (Figure 2, Table 2). Such a compensatory decrease in D was also found in cells treated with 2.5 and 10 mM HU (Table S1). Cell mass was found to be increased by about 30% and average DNA content per cell essentially unchanged. Thus, the incubation with 5 mM HU caused cells to become larger, with a higher average number of forks, but a lower DNA concentration. However, since initiation in these cells occurred at birth (age = 0) and in the control cells at age 5 minutes (at this age mass will have increased approximately 20%), the cell mass at initiation is quite similar.

The average rate of nucleotide incorporation per polymerase per second was calculated to be 790 nucleotides for the wild type cells (see Materials and Methods). In the presence of 5 mM HU this number decreased to 620 nucleotides. It is not known how the replication fork moves in the presence of 5 mM HU. It is possible that fork movement is slower simply because the nucleotide incorporation by the polymerase is slowed down because of the lower dNTP concentration. Alternatively, it is possible that the low nucleotide level in the cells is sensed and the fork therefore actively inhibited by dedicated (unknown) components. To gain more insight into how the fork speed is changed, for instance to see if there are pauses at certain chromosomal sites, a microarray experiment was undertaken. The result showed that all genes were present at exactly the same relative frequency under the two growth conditions (data not shown). This means that there are no pause sites at specific chromosomal sequences.

### The increased numbers of replication forks caused by the presence of HU were organized in an unchanged number of SeqA foci

Localization of newly replicated DNA was determined by immunofluorescence microscopy of cells immunostained with purified SeqA antiserum. The basis for this method is that SeqA specifically binds to the newly synthesized DNA following the replication forks [24] and the method can therefore be used to study the localization of replication forks in the cell. The SeqA focus distributions were almost the same in the two different situations, except for a slight increase in cells with 4 foci and a slight decrease in cells with 5 and 6 foci for the HU-treated cells compared to the untreated cells (Figure 2A and B, fourth panel). Since the former cells contained more forks, they had a higher number of forks per focus (3.3 compared to 2.5 for wild type, Table 2). This shows that there is a higher degree of fork co-localization in the HU-treated cells. The result shows that cells, when treated with HU, acquire a changed pattern of replication (longer C and more forks), but the distribution of SeqA foci does not change significantly. The result is in accordance with previous findings which indicate that the higher the number of generations spanned by C and D, the higher the number of forks accommodated in each SeqA focus [25].

The concentrations of NrdA and NrdB proteins in the cells after treatment with different concentrations of HU were determined by Western blots with antibodies against the two proteins. Increasing amounts of both proteins were found with increasing concentrations of HU. At 5 mM HU cells contained 5–10 fold more RNR than normal (data not shown). This is in accordance with earlier results where expression of *nrdAB* genes was found to be increased after HU-treatment [39]. Transcription from the *nrdA* promoter was determined by a *lacZ* reporter assay and found to be about twice that of the untreated wild type cells in the presence of 5 mM HU (data not shown). It was previously shown that the *nrdA* promoter activity is negatively regulated by the dNTP pool, i.e. a high level of dNTPs leads to low expression of RNR genes [9]. Since increased promoter activity was found in HU-treated cells, indicating a low dNTP pool, it is reasonable to assume that most of the RNR present must be inactive. The results indicate that an excess amount of inactive RNR does not affect the distribution of SeqA foci significantly.

### Reduction of dNTP levels by ectopically expressed RNR activity

**Figure 3:**
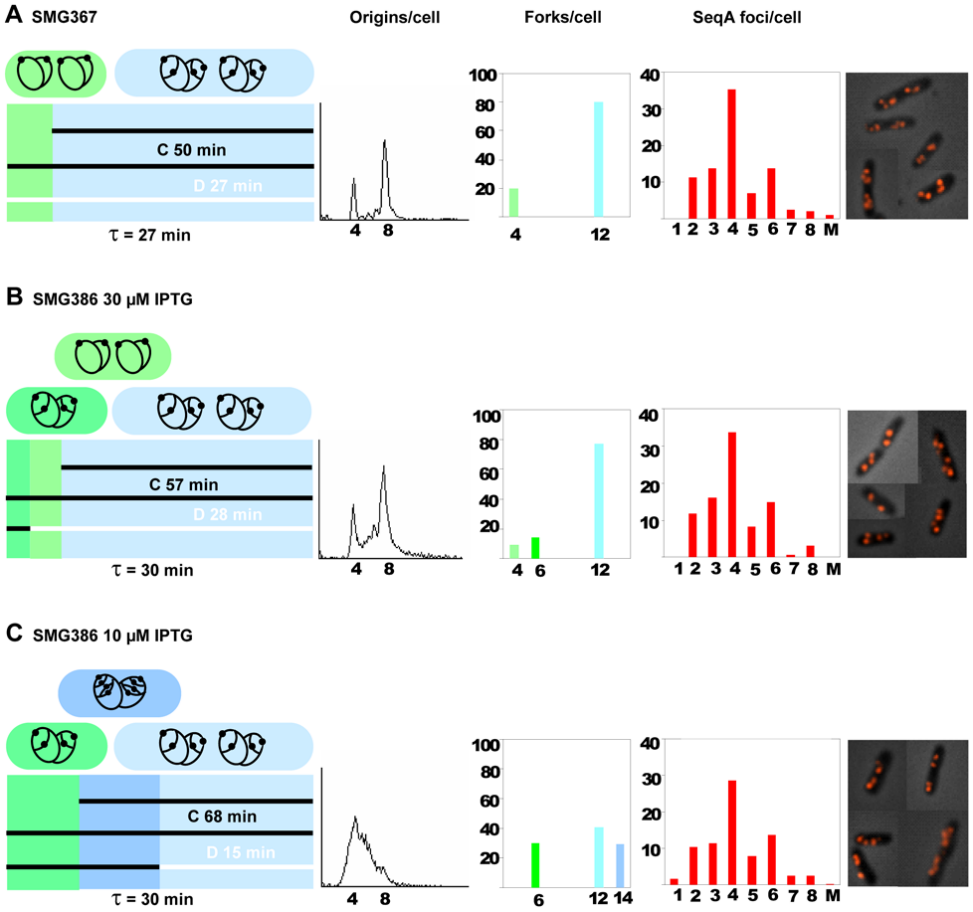
Replication patterns, fork- and SeqA focus distributions in cells with different amounts of RNR activity. Cells were grown exponentially in glu-CAA-uri medium at 37°C. A: SMG367 cells (wild type cells with empty vector; MG1655/pDSW204) grown in the presence of 20 µM IPTG. B and C: SMG386 cells (MG1655Δ*nrdAB*/pIO5) grown in the presence of 30 and 10 µM IPTG, respectively. For detailed explanations see legend to Figure 2. Fourth panel: A: 204 cells counted, the foci number of 13.6% of the cells was not determined. B: 169 cells counted, the foci number of 11.8% of the cells was not determined. C: 291 cells counted, the foci number of 21.3% of the cells was not determined.

We also investigated a situation where cells grew with low concentration of dNTPs due to a limiting amount of mutant RNR protein. The plasmid pIO5 carries the genes for a mutant RNR protein under control of an IPTG-inducible promoter (Table 1). The *nrdAB* deletion mutant, SMG386, carrying this plasmid was induced with either 10 or 30 µM IPTG. Flow cytometry analysis revealed that cells treated with rifampicin and cephalexin were unable to complete run-out replication when grown with 10 µM IPTG (Figure 3C, second panel), whereas incubation with 30 µM IPTG yielded a run-out DNA histogram comparable to that of the wild type control (Figure 3B, second panel). A reasonable explanation for these observations is that the 10 µM IPTG-dependent expression of mutant RNR proteins provides a dNTP pool that is too small for the cells to be able to finish replication after rifampicin-treatment when all mRNA synthesis stops, whereas 30 µM IPTG is sufficient to provide nucleotides also for replication run-out.

**Figure 4:**
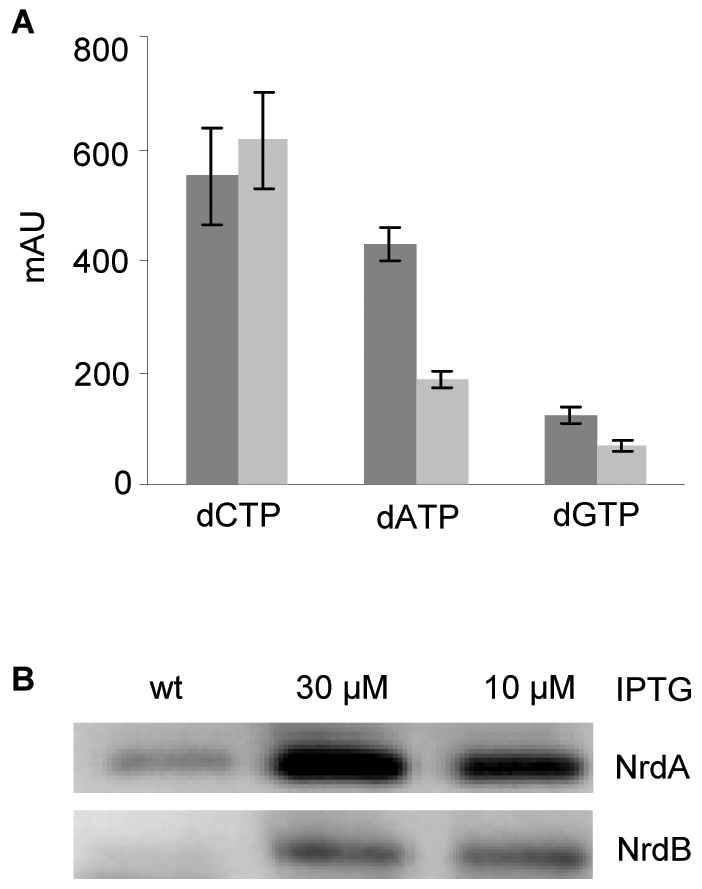
Reduced concentrations of dNTPs in cells where mutant RNR is expressed from an inducible promoter. A: Nucleotides were extracted from wild type cells and SMG386 cells grown in the presence of 10 µM IPTG and analyzed by HPLC. The value of each dNTP is given as an arbitrary unit corresponding to the area under the curve. Each bar represents the average of three independent experiments and the standard error of the mean (SEM) is shown. The level of dTTP was measured after an additional purification step using boronate affinity chromatography as explained in Materials and Methods. B: Western blot with antibodies against NrdA or NrdB proteins. Lane 1: 10 µg cell extract of SMG367 cells (wt), lane 2 and 3: 10 µg cell extracts of SMG386 cells grown in the presence of 30 or 10 µM IPTG, respectively.

In order to confirm this assumption the nucleotide concentration in the cells grown under the different conditions was measured by HPLC. In cells grown in the presence of 10 µM IPTG the amount of purines (dATP and dGTP) and dTTP was reduced approximately 2-fold compared to wild type cells whereas the concentration of dCTP was about the same as in wild type cells (Figure 4A and data not shown). No significant difference in NTP concentrations was observed between the two strains (data not shown).

Western blots with antibodies against NrdA and NrdB proteins showed an increased concentration (about 5–7 fold) of both proteins in the cells treated with 10 or 30 µM IPTG compared to wild type cells (Figure 4B), indicating that the mutant RNR protein is less active than wild type RNR protein.

In the wild type control with empty vector (SMG367) the replication pattern was essentially the same as in the wild type without plasmid (Figure 2A and 3A). Initiation of replication occurred at 4 origins early in the grandmother generation at age 4 minutes (Figure 3A). The deletion mutant carrying plasmid pIO5, had slightly increased generation times (30 instead of 27 minutes), and a similar replication pattern as wild type when grown in 30 µM IPTG (Figure 3B). When grown in 10 µM IPTG the C period was increased by 30–40%, and the D period decreased by 50–60% (Figure 3C and Table 3). The result indicates a change in replication pattern similar to that seen for HU-treated cells (Table 2), with an extention of the C period and a reduction of the D period. The average rate of nucleotide incorporation was under these circumstances 570 nucleotides per polymerase per second. The nature of the C period extension was again probed by a microarray gene dosage experiment. The relative frequency of gene copies around the chromosome was exactly the same in SMG386 grown with 10 µM IPTG as in the control, indicating that the decrease in replication fork speed is on average distributed evenly throughout the chromosome (data not shown).

**Table 3:** Cell cycle parameters of cells with limiting amounts of RNR activity.

Strain	τ^a^	DNA content^b^	Cell mass^b^	DNA/mass^b^	*oriC/terC* ^c^	C+D^d^	C^e^	D^f^	Average no of forks/cell^g^	Average no of SeqA foci/cell	Forks/focus
SMG367	27	1	1	1	3.7±0.6	77	50±6	27	10.4	4.2	2.5
SMG386 30 µM IPTG	30	1.0	0.9	1.1	3.5±0.4	85	57±6	28	10.3	4.1	2.5
SMG386 10 µM IPTG	30	0.9	1.0	0.9	4.4±0.8	83	68±8	15	10.8	4.2	2.6

a–g See legend to Table 2.

b All values are relative to SMG367.

d The C+D period of cells grown in the presence of 10 µM IPTG was obtained from the exponential DNA histogram since these cells were unable to complete run-out replication under this growth condition.

The SeqA focus distributions were found to be essentially unchanged (Figure 3, fourth panel and Table 3). The result supports the findings described above, that limiting RNR activity and low concentrations of dNTPs do not affect the distribution of SeqA foci significantly.

### Replication fork organization in a *nrdA*(ts) mutant at the permissive temperature

The third situation of limiting dNTPs we investigated was growth of the much studied temperature-sensitive *nrdA* mutant strain, E101 [40], [41]. We sequenced the *nrdA* gene and found that the mutant allele had a single missense mutation, causing a change in amino acid 89 (L89P). It has been reported that in E101 cells grown at the permissive temperature (30°C) the RNR activity is only 5% compared to that of the wild type protein, and that all activity is lost after 2 minutes incubation at the non-permissive temperature [40]. It was also found that the dNTP pool of E101 is lower than that of cells with wild type RNR protein, at the permissive temperature, and that a shift to the non-permissive temperature caused an immediate decrease in DNA synthesis with no comparable decrease in dNTP levels [42].

**Figure 5:**
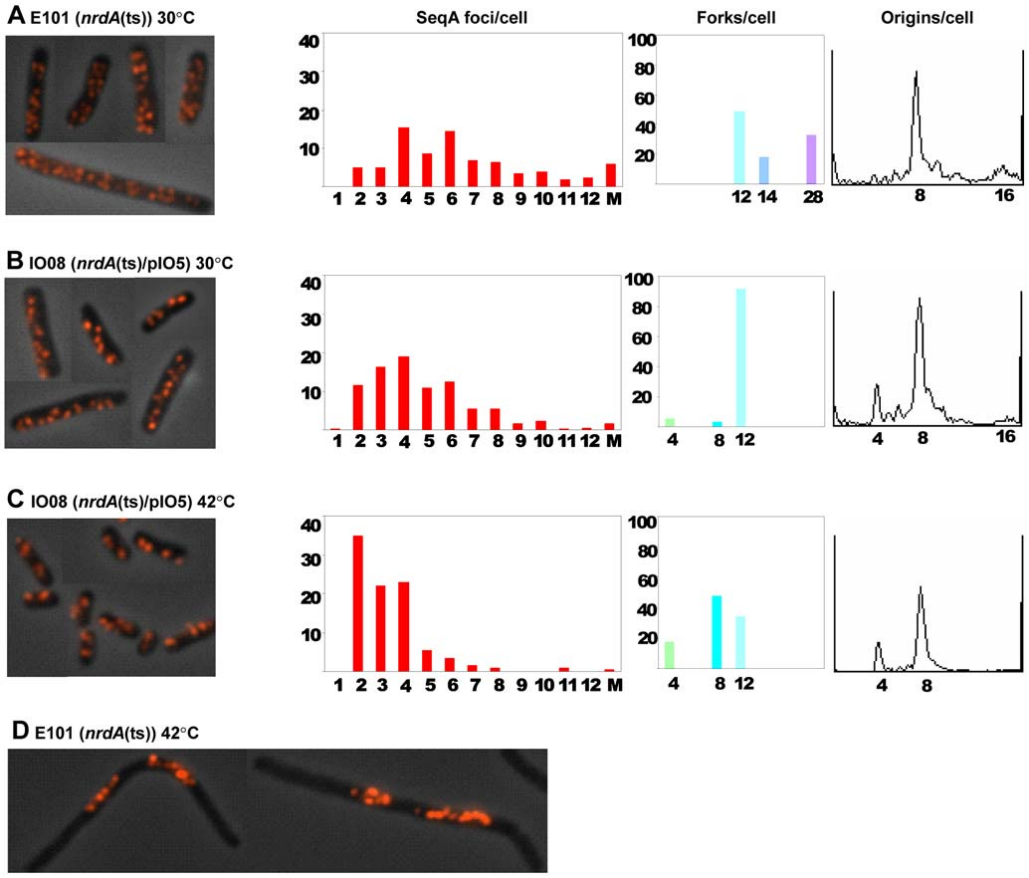
Organization of replication forks in *nrdA* (ts) mutant cells. The temperature-sensitive E101 strain (A and D) and the IO08 (E101/pIO5) strain (B and C), were grown exponentially in glu-CAA-thy medium at 30°C and at 42°C for the equivalent of three generations after temperature shift-up. IO08 cells grew in the presence of 30 µM IPTG. First panel: micrographs of fixed cells immunostained with SeqA antibody. Second panel: SeqA foci distribution where the percentages of cells are plotted against the number of foci per cell. Third panel: replication fork distribution where the percentages of cells are plotted against the number of replication forks per cell. Fourth panel: DNA histogram of cells treated with rifampicin and cephalexin. A: 450 cells counted, the foci number of 22% of the cells was not determined, B: 407 cells counted, 13% not determined, C: 241 cells counted, 8% not determined. See Figure S2 for detailed description of replication patterns.

E101 cells were grown exponentially in glu-CAA-thy medium at the permissive temperature (30°C). The C+D period was found to span four generations (cells had 8 or 16 origins, Figure 5A, see Figure S2 for detailed description of the replication pattern) and the C period was calculated to be 132 minutes (Table 4) and thus lasting about 2.5 generations compared to 1.8 in MG1655 wild type cells (Table 2). This led to a high number of replication forks (17.6 forks on average, Figure 5A, Table 4). The average number of SeqA foci was also found to be high (6.8 foci on average, Table 4). Since both the numbers of replication forks and SeqA foci were high, the numbers of forks per focus was found to be relatively unchanged (2.6 forks/focus, Table 4).

**Table 4:** Cell cycle parameters of the *nrdA* (ts) mutant strain (E101) and the IO08 (E101/pIO5) strain.

Strain	τ^a^	DNA content^b^	Cell mass^b^	DNA/mass^b^	*oriC/terC* ^c^	C+D^d^	C^e^	D^f^	Average no of forks/cell^g^	Average no of SeqA foci/cell	Forks/focus
E101^h^	52	1.5	2.4	0.6	5.6±1.0	177	132±14	45	17.6	6.8	2.6
IO08 30°C	50	1.4	1.9	0.7	3.6±0.8	148	96±15	52	11.5	4.9	2.3
IO08 42°C	30	1.4	1.5	0.9	2.6±0.3	86	39±6	47	8.6	3.3	2.6

a–g See legend to Table 2.

b All values are relative to MG1655 grown without HU in Table 2.

h Experiments with E101 containing empty vector (E101/pDSW204) gave the same average DNA content and cell mass as well as the same generation time, the same number of origins after run-out replication and the same SeqA focus distribution as E101 (data not shown).

The cell mass and DNA content of the mutant E101 cells were highly increased and the DNA concentration was decreased relative to MG1655 cells (Table 4). Most changes were similar to those found for treatment with HU (Table 2). The main difference compared with HU-treated cells was the high increase in mass and the high number of SeqA foci per cell. Under all conditions tested above, the SeqA concentration remained the same (data not shown).

The cell cycle parameters of E101 could be normalized by ectopically supplied RNR activity (Figure 5B and C, Table 4). After a shift to the non-permissive temperature (42°C) and growth for the equivalent of 3 generations, the *nrdA* (ts) mutant cells (E101) became filamentous and the formation of discrete SeqA foci was lost (Figure 5D).

### Initiation of replication is unaffected by changes in elongation rate

In the experiment with ectopically supplied mutant RNR (strain SMG386), the extension of C was compensated by a reduction of D such that the time point of initiation was essentially unchanged because C+D was unchanged. In the experiment where wild type cells were grown in the presence of 5 mM HU, the reduction in D was less than the increase in C, and in the experiment with the severely impaired, temperature-sensitive mutant RNR (strain E101) the D period was almost unchanged. In the latter two situations C+D was increased and therefore the time point of initiation was moved to earlier in the cell cycle. At first glance this seems strange. How can the initiation complex be assembled earlier than normal? This can, however, be explained if we also consider cell mass. In the latter two experiments it was found that cell mass was increased, i.e. cell division occurred at a larger mass than normal. Thus, it is likely that the requirements for initiation components have not changed, and that the initiation mass is not significantly changed. The cells are just growing as larger cells (and will reach a required “initiation mass” at an earlier point in life). The results thus indicate that the decrease in cellular dNTPs mainly affects replication fork elongation and cell division, and not initiation of replication.

### The phenotype of the *nrdA* (ts) mutant is not caused by replication fork stalling

**Figure 6:**
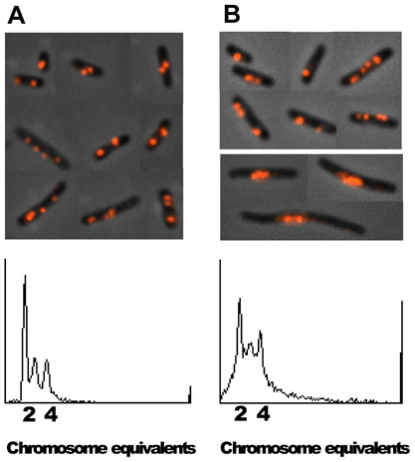
SeqA focus patterns in cells undergoing replication fork stalling and reversal. *dnaN159* (ts) mutant cells were grown exponentially in glu-CAA-uri medium at permissive (30°C) (A) and semi-permissive temperature (37°C) (B). Fixed cells were immunostained with antibody against SeqA. The DNA histogram of the same cells treated with rifampicin and cephalexin is shown at the bottom of each panel. The experiment was repeated twice (with the same results).

It has previously been reported that replication forks in the *nrdA* (ts) mutant frequently stall and are recovered by the replication fork reversal process [13], [43]. We considered it possible that the phenotype of E101 (many forks and foci) could be due to the replication fork stalling. In order to find out how SeqA foci appear in cells dealing with stalled replication forks, the *dnaN159* (ts) mutant encoding a mutant β-subunit of the DNA polymerase III holoenzyme, was studied. The replication forks in this mutant undergo arrest when the cells are grown at the semi-permissive temperature (37°C) [44]. Flow cytometry analysis confirmed that the *dnaN159* (ts) cells grown at 37°C had problems with completion of replication run-out presumably due to stalling of replication forks (Figure 6B, bottom panel). Immunofluorescence microscopy revealed that approximately 5% of the cells were enlarged with small, central nuclei (Figure 6B, upper panel). This phenotype was not observed when the cells were grown at the permissive temperature (Figure 6A). The enlarged cells had lost the ability to form discrete SeqA foci. Instead the SeqA protein was found co-localized with the DNA in the middle of the cells (Figure 6B and data not shown). These cells resemble E101 cells at non-permissive temperature (Figure 5D), and may represent either dying cells with stalled forks that could not be successfully repaired or cells that have elicited the SOS response and are going through a specific repair program (see Discussion). About 1% of the E101 mutant cells also showed this phenotype at the permissive temperature (data not shown). Also SMG386 cells grown in the presence of amounts of IPTG lower than 10 µM acquired this appearance (data not shown). The results indicate that a decrease in the dNTP concentrations below a certain level leads to cessation of growth with elongated cells and compact nuclei with relatively uniform SeqA immunostaining across the nuclei. A normal pattern of SeqA structures was observed in most of the *dnaN159* (ts) mutant cells. This indicates that in cells where the stalled forks are reversed and repaired, the SeqA structure formation is unchanged. Hence, the presence of an increased number of stalled forks is probably not the reason for the phenotype observed in the *nrdA* (ts) mutant at the permissive temperature.

### Repair of forks after UV damage is performed in a compact nucleoid structure without distinct SeqA foci

**Figure 7:**
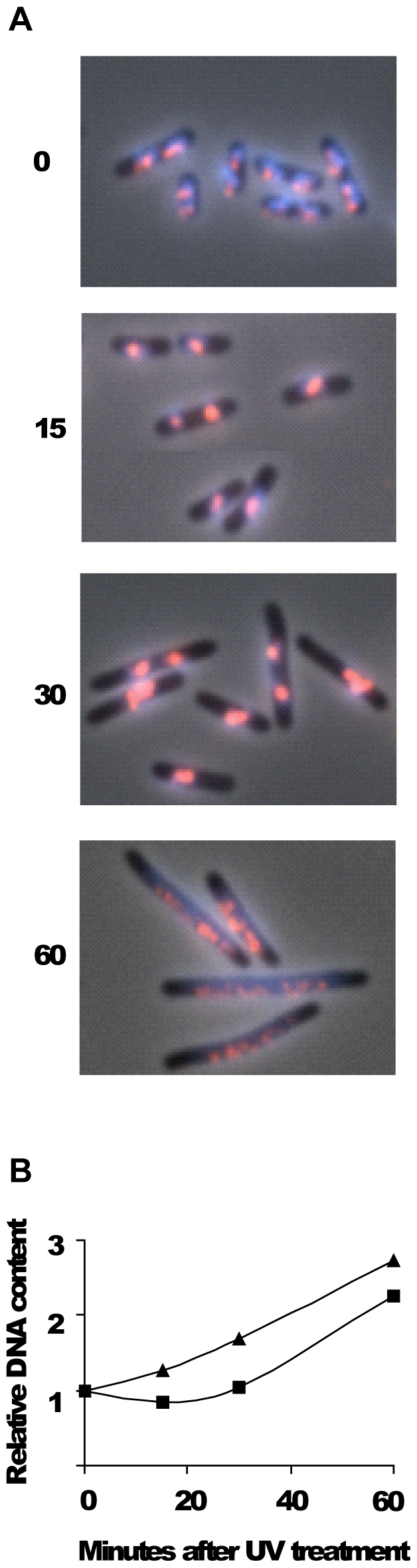
Localization of DNA and SeqA in cells irradiated with UV. MG1655 wild type cells were grown exponentially in glu-CAA-uri medium at 37°C to OD_450_ = 0.15 and then irradiated with UV (50 J/m^2^). Samples were taken at the time points indicated and fixed in 70% ethanol. A: Cells were immunostained with antibody against SeqA (red) and the DNA was stained with Hoechst 33258 (blue). The two fluorescence images are shown merged with the phase-contrast image. B: Relative DNA content per cell obtained from flow cytometry analysis as a function of time after UV treatment. One portion of exponentially growing cells (OD_450_ = 0.15) was exposed to UV while another portion was mock-treated, then 10 µg/ml cephalexin was added to both cultures and samples taken at the time indicated. ▴: mock-treated cells, ▪: irradiated cells. The data in panel B are the average of two independent experiments. Samples in panel A were not treated with cephalexin.

To find out more about what a SeqA focus looks like in a cell where the forks are stalled, we analyzed cells that had received enough damage to stall most forks in most cells. When cells are irradiated with UV, most DNA synthesis stops for at least 15–20 minutes before it continues again when the replication forks have been repaired [45]. Undamaged origins, however, continue to initiate at the normal rate [45]. MG1655 wild type cells were grown in glu-CAA-uri medium at 37°C to early exponential phase and irradiated with 50 J/m^2^ of UV light. Samples for flow cytometry and microscopy were taken at different time points after UV irradiation. Staining with Hoechst showed that the DNA became highly compact and apparently took up much less space in each cell (compare 15 and 30 minute with 0 minute time points, Figure 7A). The immunostaining of SeqA covered most of the compact Hoechst-stained nucleoids. Flow cytometry analysis showed that DNA synthesis was delayed at least 30 minutes after UV irradiation (Figure 7B). After 60 minutes the DNA was again more normally spread out in the cells and small, discrete SeqA structures appeared (Figure 7A). This was also the case after 90 minutes (data not shown). This result indicates that the normal SeqA focus distribution is lost soon after irradiation when most forks are stalled and then slowly reappears again when forks are repaired and replication continued. The SeqA focus distribution was, however, not normal after 60 and 90 minutes (compare Figure 2A and 7A). Since cell division is still inhibited at these time points it could be that the cells are not segregating their DNA in the usual manner. This result indicates that normal segregation is required for normal SeqA focus formation. The apparently unspecific binding of SeqA that appears after UV irradiation may represent binding of SeqA protein also to fully methylated DNA. Although undamaged origins are reported to initiate as normal after irradiation [45], and some hemimethylated DNA should be present in the irradiated cells, the results from Figure 7 show that the SeqA protein seems to interact with most of the compacted repair site DNA. This indicates that normal amounts of hemimethylated DNA may not be present during the SOS response. Alternatively, the DNA may be obscured and not accessible to SeqA binding (see below for further discussion).

## Discussion

### A limitation in cellular dNTPs mainly affects replication fork elongation and cell division, and not initiation of replication

In this work we find that replication forks can be made to move more slowly than normal, if a moderate limitation of nucleotides is created. Whereas a severe limitation in dNTPs leads to frequent replication fork stalling (and probably reversal) and dependence on recombination enzymes, the mild limitation studied here, led to a controlled slowing of replication forks. Each polymerase went from incorporating the normal, on average 800 nucleotides per second, to incorporating on average 600 nucleotides per second. The relative frequency of gene copies around the chromosome remained the same showing that the change in fork speed was evenly distributed.

Measurements of cell mass and DNA concentration showed that the mass at the time of initiation was roughly unchanged in both the experiments with relatively well functioning RNR, and whereas average cell mass was about 30% increased in the HU experiment, it was unchanged in the experiment with ectopically regulated expression of RNR. Thus, the considerable change in replication elongation rate did not lead to a significant change in the regulation of initiation, but did (in one case) lead to changes in the control of cell division. The “delay” in cell division which makes the HU-treated cells grow with a 30% larger mass, probably is necessary for completion of chromosome replication and segregation. It is known that checkpoints are activated to delay cell division if chromosome replication and segregation is incomplete (e.g. the SOS response). In the experiment with ectopically regulated expression of mutant RNR the growth rate was 10% increased. This is possibly a reason why these cells were able to accommodate the extended C period without delaying cell division.

In cells with the poorly functioning mutant NrdA1 protein similar effects were seen. Cells grew with a highly extended C period and a larger mass. These phenotypes were reversed upon complementation.

### No evidence was found for replication hyperstructures affected by RNR activity

During bacteriophage replication, structures form that contain both nucleotide synthesizing enzymes and replication enzymes. These structures presumably increase the efficiency of viral replication [1], [4], [5]. It has been suggested that the formation of SeqA structures at the chromosomal replication forks might function as scaffolds for similar kinds of enzyme assemblies (replication hyperstructures) [2], [21]. It has also been shown that the normal distribution of SeqA foci appeared to fall apart if the synthesis of thymine nucleotides was limited [24]. We find here that changes in the activity of ribonucleotide reductase do not compromise the normal distribution of SeqA foci. We also find that cells with a severely impaired RNR are capable of replication fork co-localization. The average number of forks per focus was not significantly changed although the average number of SeqA foci per cell was increased about 70%. These cells suffer from more stalling and reversal of replication forks than normal [13]. Thus, the results presented here, indicate that replication fork reversal does not affect the distribution of SeqA foci.

### SeqA foci could not be distinguished in the compact repair structure assembled after UV damage

We find here that SeqA protein binds relatively unspecifically to a very compact nucleoid for at least 30 minutes following UV irradiation. It is possible that the replication forks experience prolonged stalling and that most of the new, hemimethylated DNA becomes fully methylated, and that SeqA then must bind fully methylated DNA (i.e. all GATC sites on the chromosome with equal preference).

The stress response induced by UV irradiation led to a dramatic change in the appearance of the nucleoid indicating that a major reorganization occurs. It is possible that the SOS response directs the generation of a “repair domain” in which repair enzymes are organized and damaged DNA and forks may be processed. Studies by Rudolph and co-workers indicate that the RecFOR-mediated loading of RecA at stalled forks, protects and stabilizes them after UV irradiation [46]. The microscopy images shown in their Figure 1 [46] indicate a compaction of nucleoids in wild type cells during the first part of the SOS response, similar to that observed here at 15 and 30 minutes (Figure 7A). This DNA compaction seems to be absent in their images of a *recO* mutant [46]. It is thus possible that RecFOR and RecA organize the DNA into a specialized structure suited for efficient DNA repair, and if so, the compact nucleoid in the middle of the cell could represent such a repair factory. If much of the DNA in this situation is covered by RecA protein, this may affect how the SeqA protein binds and whether its binding sites are accessible or not.

Also, 5% of the *dnaN159* (ts) cells grown at semipermissive temperature, 1% of the E101 mutant cells, and SMG386 cells grown with less than 10 µM IPTG, were found to be elongated and contain small central nucleoids. It is thus possible that an SOS response and the suggested RecO-dependent repair structure were induced also in these cells.

## Supporting Information

Figure S1 Method of cell cycle parameter calculation
(0.11 MB PDF)

Figure S2 Replication patterns of nrdA mutant cells
(0.05 MB PDF)

Table S1 Cell cycle parameters of cells grown with HU
(0.05 MB PDF)
