# Supplementary material for: A Reduction in Ribonucleotide Reductase Activity Slows Down the Chromosome Replication Fork but Does Not Change Its Localization
Source: PLoS One. 2009 Oct 28;4(10):e7617. doi: 10.1371/journal.pone.0007617 (PMC2773459; doi:10.1371/journal.pone.0007617)
Supplement: Figure S1 — Method of cell cycle parameter calculation (0.11 MB PDF) [file pone.0007617.s001.pdf]

Figure S1 Cell cycle analysis.

A

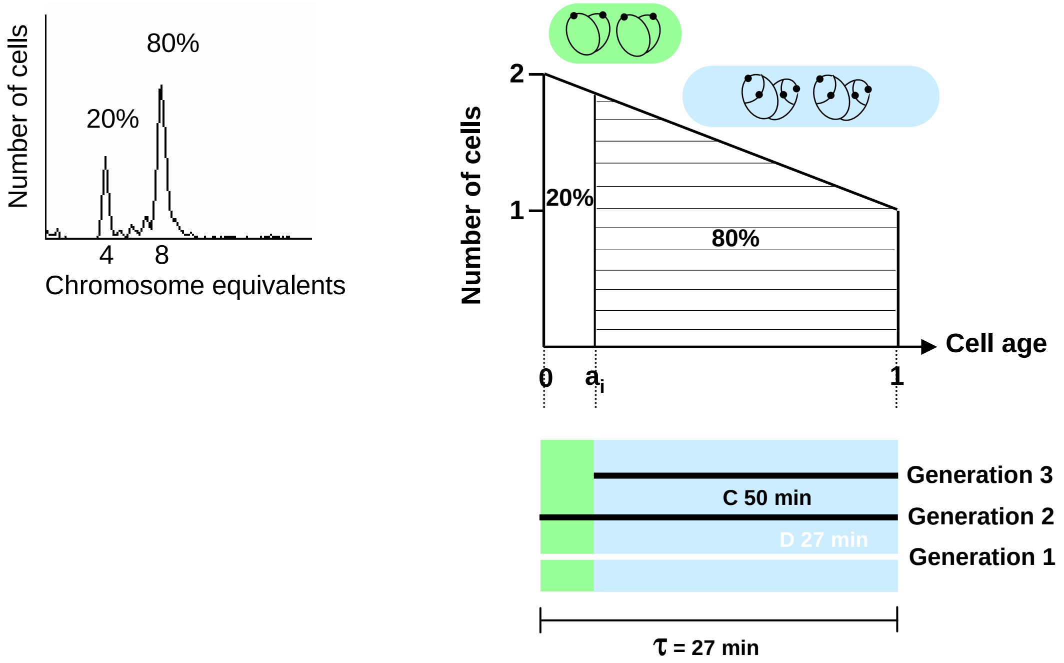

B

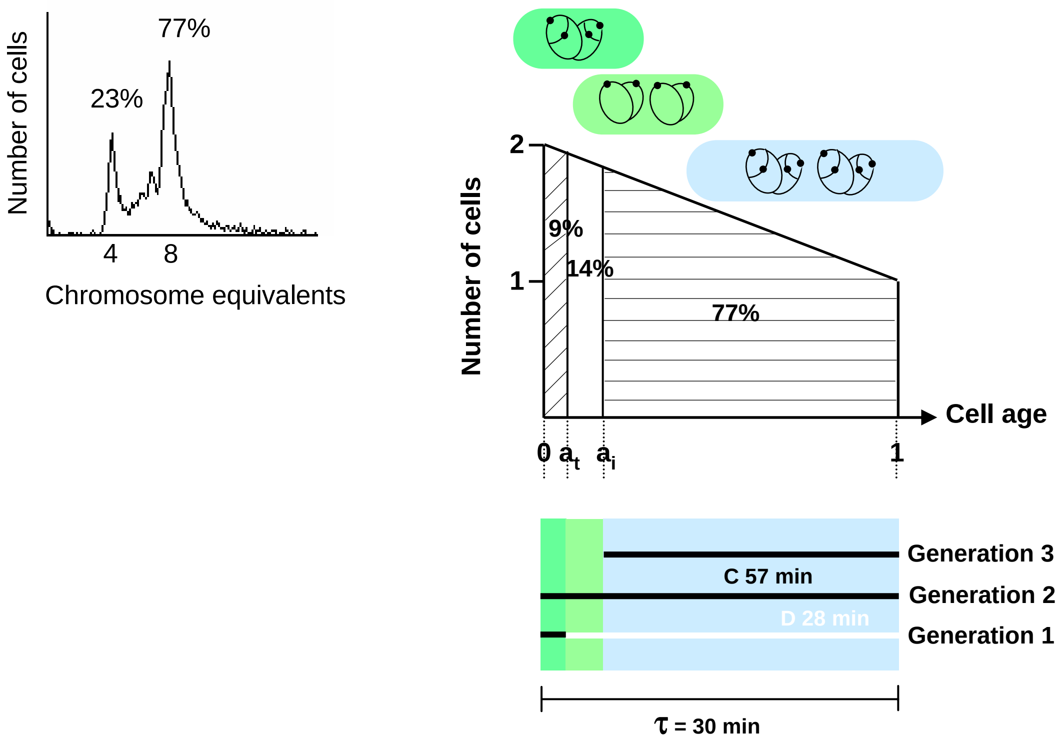

SMG367 and SMG386 cells grown in the presence of 30 μM IPTG (from Figure 3A and B, respectively) are used as examples of cell cycles where initiation occurs before (A) or after (B) termination. In A termination coincides with cell division. Rifampicin and cephalexin inhibit initiation of replication and cell division, respectively, but allow the completion of ongoing replication. From the rifampicin run-out histogram the fraction of cells that had not initiated replication at the time of drug action can be found. In A, this fraction corresponds to 20% of the cells (4-origin cells) while 80% of the cells (8-origin cells) had initiated replication at the time of drug action. Since initiation occurs in cells containing 4 origins, we infer that the replication cycle spans 3 generations. The cells are in balanced, steady state growth (all cells have the same replication and division pattern), so it is possible to calculate an average initiation age (a_i), at which a cell initiates the 4 origins, and an average termination age (a_t), for cells in this culture. To find the initiation age, we use the information we have about how many of the cells have 4 origins (20%), combined with the age distribution of an exponentially growing population (A, right panel). (In this figure the exponential curve is shown approximated by a linear one. This approximation is slight and is included here to show that it is possible to estimate parameters graphically without the use of the formulas). The fraction of cells with ages from 0 to a_i (F) is the fraction of cells (20%) that had not initiated. This fraction of the population is given either by $F= 2 – 2^{(1-ai)}$ where a_i is the initiation age given as a fraction of a generation, or by $F = 2 - 2^{((\tau-\alpha i)/\tau)}$ where α_i is the initiation age given in minutes and τ is the generation time in minutes (27 min). Using this formula we find that initiation occurs after 4 min, at age 0.148 (A, right panel). Since we know that the C+D period spans 3 generations we find C+D by subtracting 4 min from 3 x 27 min, i.e. C+D=3 (generations) x 27 min (τ) – 4 min (α_i) = 77 min. The C period, the time required for a round of replication, was

determined by the formula *oriC*/*terC*=$2^{C/\tau}$, where the *oriC*/*terC* ratio was measured by Southern blot and quantitative PCR as described in Materials and Methods. The C period was found to be 50 min (horizontal black lines in the schematic diagram of the cell cycle in A, right panel). With this information, i.e. how much of C+D is taken up by C, we can find the D period and the age at termination. C takes up 23 min of generation 3 and 27 min of generation 2. Thus, termination occurs exactly at cell division, or at age 1, and D = 77 min (C+D) – 50 min (C) = 27 min (horizontal white line in A, right panel). Since termination of the oldest forks occurs at cell division no cells with 6 forks are found in this cell population. The fraction of cells with ages 0-a_i corresponds to the fraction of cells that had not initiated at the time of drug action, i.e. the fraction of 4-origin cells (20%, blank area under the age distribution curve). The fraction of cells with ages between a_i-1 corresponds to the fraction of cells that had initiated at the time of drug action, i.e. the fraction of 8-origin cells (80%, striped area under the age distribution curve). The number of replication forks and origins in the different phases of the cell cycle is shown in schematic cells (light green and blue rods) with chromosomes (black lines) and origins (black dots) (A, right panel). Thus, 20% of the cells in the population had 4 replication forks and 80% had 12 replication forks. From this information the average number of forks per cell was found to be 10.4.

In B, the rifampicin run-out DNA histogram shows that 30 μM IPTG is on the boarder of being sufficient for supplying nucleotides and therefore run-out is not absolutely complete. It was estimated that 23% of the cells had 4 origins and 77% 8 origins. As in A, initiation occurs in cells containing 4 origins and the replication cycle therefore spans 3 generations. From the same formulas as used in example A, the initiation age is found to be 5 min while the C+D, the C and D periods are found to be 85, 57 and 28 min, respectively. Since termination

(α_t) occurs at 2 min, the fraction of cells with ages 0-a_t is 9% (hatched area under the age distribution curve, $F = 2 - 2^{((\tau-\alpha t)/\tau)}$). The fraction of cells with ages a_t-a_i is 23% - 9% = 14% (blank area under the age distribution curve). The fraction of cells with ages a_i – 1 corresponds to the fraction of cells that had initiated at the time of drug action, i.e. the fraction of 8-origin cells (77%, striped area under the age distribution curve). Since 9% of the cells in the population had 6 replication forks, 14% had 4 replication forks and 77% had 12 replication forks, the average number of forks per cell was found to be 10.3.

Calculation of theoretical DNA distributions:

For the culture in A the DNA contents of cells at ages 0, a_i and 1 were calculated by the formulas $2+2(\tau-\alpha_i)/C$, $2+2\tau/C$ and $4+4(\tau-\alpha_i)/C$, respectively (confer with schematic drawings of cells for numbers of fully and partially replicated chromosomes). The following DNA contents were found: 2.92, 3.08 and 5.84 chromosome equivalents. The theoretical DNA distribution was generated by forming exponential distributions containing 20% of the cells within the DNA interval 2.92-3.08 and 80% of the cells within the interval 3.08-5.84. For the culture in B, the DNA contents of cells at the ages 0, a_t, a_i, and 1 were calculated by the formulas $1+(2\tau-\alpha_i)/C+2(\tau-\alpha_i)/C$, $2+2(\tau-\alpha_i+\alpha_t)/C$, $2+2\tau/C$, $2+2(2\tau-\alpha_i)/C+4(\tau-\alpha_i)/C$, respectively, and found to be 2.84, 2.95, 3.05 and 5.68 chromosome equivalents. The theoretical DNA distribution was generated by forming exponential distributions containing 9% of the cells within the DNA interval 2.84-2.95, 14% of the cells within the interval 2.95-3.05 and 77% of the cells within the interval 3.05-5.68. The theoretical distributions were compared to the experimental exponential distribution to verify that the cell cycle parameters found by the Southern blot analysis, quantitative PCR and rifampicin run-out measurements were correct.
