# Supplementary material for: A Reduction in Ribonucleotide Reductase Activity Slows Down the Chromosome Replication Fork but Does Not Change Its Localization
Source: PLoS One. 2009 Oct 28;4(10):e7617. doi: 10.1371/journal.pone.0007617 (PMC2773459; doi:10.1371/journal.pone.0007617)
Supplement: Figure S2 — Replication patterns of nrdA mutant cells (0.05 MB PDF) [file pone.0007617.s002.pdf]

Figure S2 Replication patterns of strains E101 and IO08 (E101/pIO5).

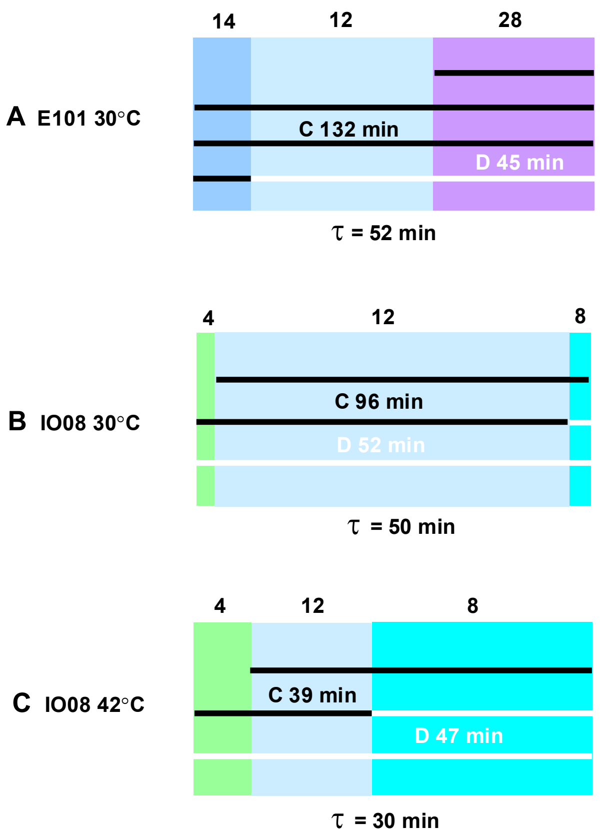

See legend to Figure 2 for detailed explanations. The fork numbers corresponding to the three different stages of the cell cycle are given above each diagram and the colors used are the same as the ones used in Figure 5 (third panel).
