# Supplementary material for: A Reduction in Ribonucleotide Reductase Activity Slows Down the Chromosome Replication Fork but Does Not Change Its Localization
Source: PLoS One. 2009 Oct 28;4(10):e7617. doi: 10.1371/journal.pone.0007617 (PMC2773459; doi:10.1371/journal.pone.0007617)
Supplement: Table S1 — Cell cycle parameters of cells grown with HU (0.05 MB PDF) [file pone.0007617.s003.pdf]

Table S1 Cell cycle parameters of cells grown with the indicated concentrations of HU.

mM HU	τ (min)	C	D	C+D
0	27	49	27	76
2.5	27	56	23	79
5	27	62	19	81
10	30	82	15	97

The C+D period was determined from the initiation age, generation time and number of generations spanned by C+D as explained in legend to Figure S1. For cells grown without or in the presence of 5 mM HU C periods were obtained from Southern blot analysis and quantitative PCR, while the D periods were found from (C+D)-C. For cells grown in the presence of 2.5 and 10 mM HU, the C and D periods were obtained from theoretical simulation where the values of C and D that gave the best fit to the exponential histogram were chosen.
